# Successful excision of a giant cervical mediastinal goiter by cervical collar incision and a thoracoscopic approach: a case report

**DOI:** 10.1186/s44215-023-00058-x

**Published:** 2023-07-20

**Authors:** Junichi Morimoto, Takahiro Yamanaka, Jotaro Yusa, Takahiro Ochi, Taisuke Kaiho, Kota Ohashi, Yuki Shiina, Yuki Sata, Takahide Toyoda, Atsushi Hata, Takayoshi Yamamoto, Yuichi Sakairi, Hironobu Wada, Hidemi Suzuki, Takahiro Nakajima, Ichiro Yoshino

**Affiliations:** Department of General Thoracic Surgery, Chiba University Graduate School of Medicine, Chiba, 260-8670 Japan

**Keywords:** Adenomatous goiter, Cervicomediastinal goiter, Cervical collar incision, Giant goiter

## Abstract

**Background:**

Giant cervicomediastinal goiter extending to the bifurcation of the trachea mostly requires median sternotomy in addition to a cervical collar incision for resection. Sternotomy provides a good operative field, although it is one of the most invasive thoracic approaches. We herein report a case of giant cervicomediastinal goiter resected by a less-invasive and highly effective method with a thoracoscopic and cervical approach.

**Case presentation:**

A 71-year-old Japanese woman with giant cervicomediastinal goiter extending to the bifurcation of the trachea was introduced and admitted to our hospital with dyspnea. Chest computed tomography showed tumor-induced airway narrowing due to the giant goiter of the right side of the thyroid lobe. We safely performed resection of the giant goiter through a cervical collar incision and thoracoscopic approach, and this combined approach contributed to the early discharge of the patient from the hospital.

**Conclusion:**

A combined approach of cervical collar incision and thoracoscopy is useful for resection of giant goiter.

## Background

Mediastinal goiter is often asymptomatic. However, immediate surgical resection is necessary for cases with symptom of dysphagia due to esophageal pressure, airway-related conditions, such as feelings of suffocation from upper respiratory tract pressure, and feelings of dyspnea. Among cases of mediastinal goiter, 95% are considered resectable only by a cervical approach [1]. However, cases of giant cervicomediastinal goiter extending to the bifurcation of the trachea typically require median sternotomy for safe resection. With sternotomy, we are able to obtain a good operating field. However, this approach also increases the risk of complications, such as bleeding, infection, dysraphism of the sternum, and postoperative pain.

We herein report a case of giant cervicomediastinal goiter resected by a less-invasive and highly effective method with a thoracoscopic and cervical approach.

## Case presentation

A 71-year-old Japanese woman was admitted to the hospital with a chief complaint of dyspnea. Physical examinations showed her oxygen saturation to be 95% on room air. She was 149 cm tall and 62 kg in weight with a body mass index of 27.9. Her electrocardiogram was normal with a sinus rhythm. Her thyroid function, liver function, renal function, and respiratory function were normal.

**Fig. 1:**
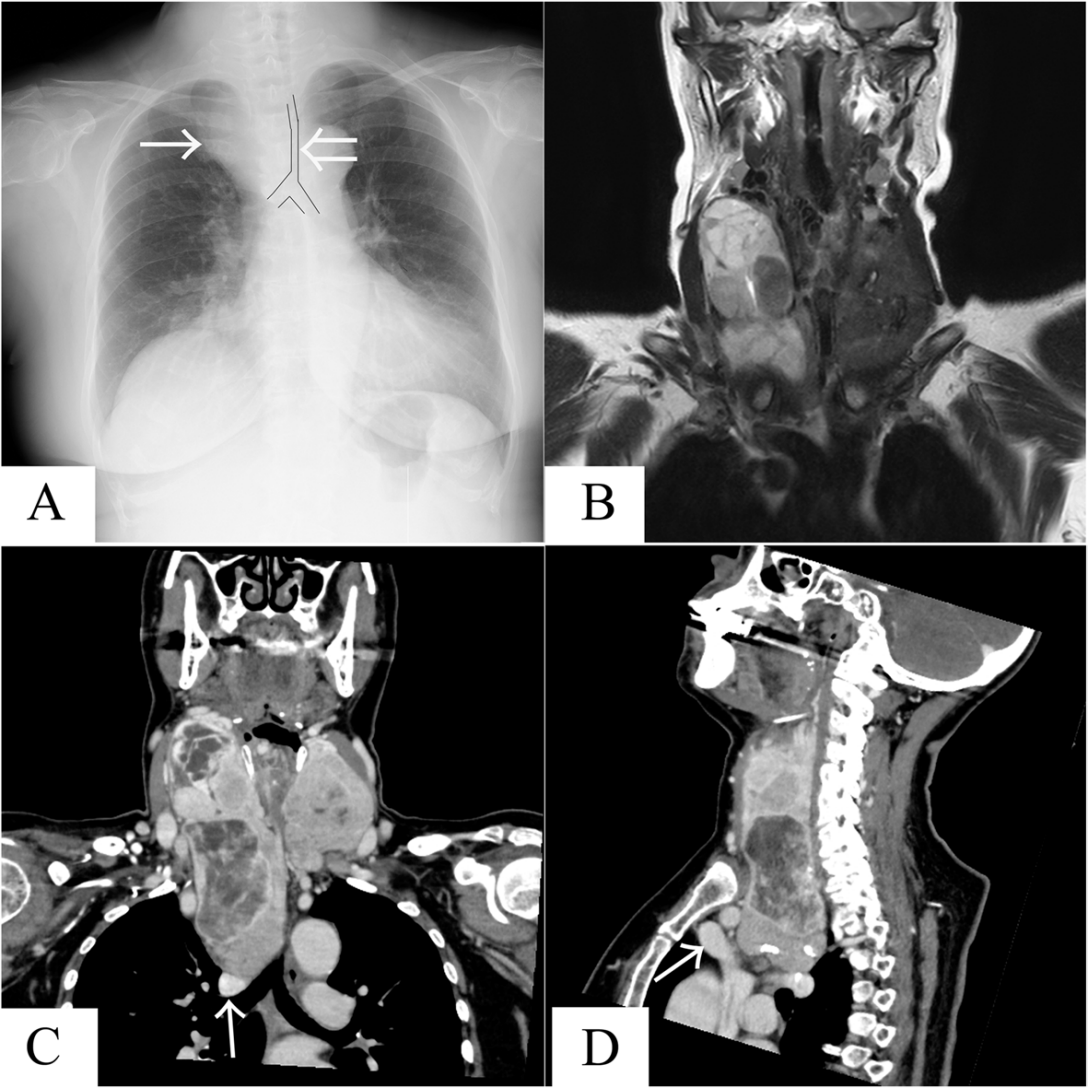
Image findings at admission. **A** Chest X-ray revealed the tracheal stenosis (large arrow) and deviation to the left side (small arrow). **B** Enhanced magnetic resonance imaging of the neck and chest showed a giant goiter with heterogeneous contrast growing in both thyroid lobes, and the trachea was pressed by the goiter and had become narrowed. **C** Coronal section of enhanced computed tomography of the neck and chest showed that caudal edge of the right goiter extended along the trachea just cranial to the azygos vein (small arrow) with no invasion. **D** A sagittal section of enhanced computed tomography showed that the goiter was not invading the right brachiocephalic vein (small arrow)

Chest X-ray showed tracheal stenosis and deviation to the left side (Fig. 1A). Enhanced computed tomography of the neck and chest showed a giant goiter contrasting heterogeneously in both thyroid lobes growing at the tracheal bifurcation level of the middle mediastinum, behind the brachiocephalic artery. The trachea was pressed by the goiter and had become narrowed (Fig. 1B). The size of the right thyroid lobe was 153 × 64 × 55 mm, and that of the left lobe was 76 × 49 × 40 mm. Preoperative contrast-enhanced computed tomography (CT) showed that caudal edge of the right goiter extended along the trachea just cranial to the azygos vein with no invasion to the surrounding structure (Fig. 1C and D). We considered the site of the goiter’s extension to coincide with the site of the dissection of the superior mediastinum and suspected that it could be dissected under thoracoscopic surgery.

Aspiration cytology and a biopsy were done under echo guidance, with results showing no malignancy. Therefore, the lesion was diagnosed as a benign adenomatous goiter.

**Fig. 2:**
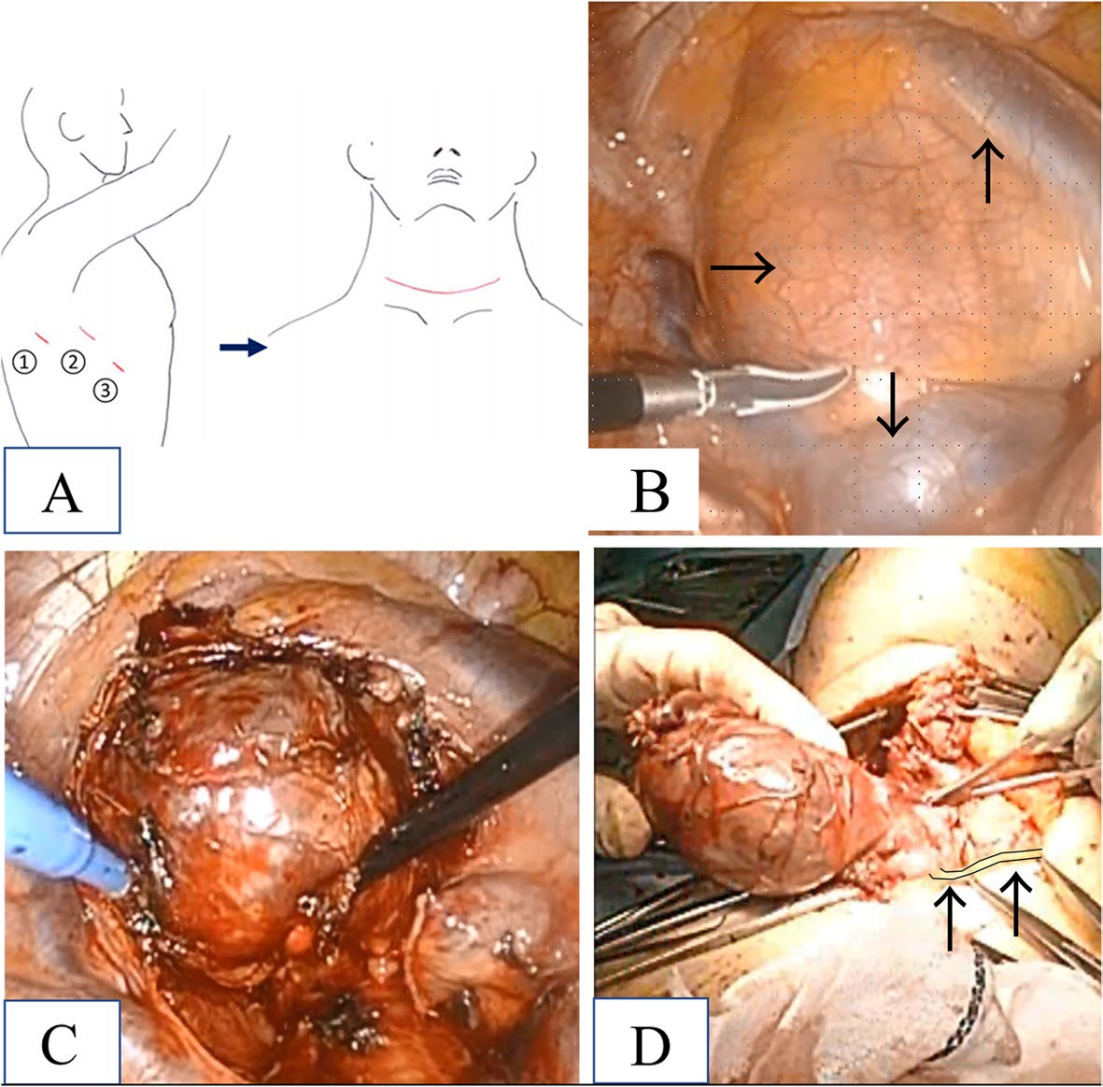
Perioperative findings. **A** The location of the thoracic port and neck incision line. (1) Seventh intercostal space at the posterior axillary line. (2) Fifth intercostal space at the midaxillary line. (3) Fifth intercostal space at the anterior-axillary line. **B** The tumor (right arrow) was located behind the superior vena cava (upward arrow), and the bottom end was behind the azygos vein (downward arrow). **C** The tumor was dissected from the superior vena cava, trachea, and azygos vein (transected). **D** The tumor of the right adenomatous goiter was resected via the neck incision. The recurrent laryngeal nerve was preserved, but it was hyperextended by goiters (two arrows)

To resolve the dyspnea, she was referred to our hospital for goiter resection. The surgery required an approach from two routes. One involved a thoracic incision by a thoracic surgeon, and another involved a neck incision by an otolaryngologist. In the preoperative conference, we decided to dissect the goiter from the thoracic cavity side first and then resect it from the cervical incision, as the caudal side of the goiter was thinner than the cranial side, and we thought it would be easier to pull it out from the neck incision. Since the cranial side of the brachiocephalic artery and vein was able to be dissected from the neck by otolaryngologists, we decided to dissect the dorsal side of the brachiocephalic artery and vein as the upper limit. We performed thoracoscopic surgery with the patient under general anesthesia and single-lung ventilation with 6.5-mm spiral tube blocking the right main bronchus by a bronchial blocker balloon. We placed the patient in the left lateral decubitus position, and three ports with 2-cm incisions were placed (one each) at the 5th intercostal space on the midaxillary line, anterior-axillary line, and 7th intercostal space on the posterior axillary line (Fig. 2A). We inserted a thoracoscope through the 5th intercostal space at the mid- or anterior axillary line and energy devices or forceps through the 7th intercostal space at the posterior axillary line or 5th intercostal space at the anterior axillary line. This was because the angle of the forceps when manipulating the upper mediastinum facilitated operations, and the back side of the tumor was easier to see when we dissected the goiter from the trachea, superior vena cava, and vertebra. The tumor was located behind superior vena cava, and the bottom end was located behind the azygos vein (Fig. 2B). Dissection of the lateral edge of the tumor was performed using LigaSure Maryland (Covidien, Mansfield, MA, USA) running along the edge of the superior vena cava, azygos vein, and posterior edge of the tumor. Next, we performed transection of the azygos vein with automatic suture instruments. We then exfoliated the tumor from the trachea, moving from the caudalis towards the head, up to the height of the brachiocephalic trunk on the head side (Fig. 2C). The goiter in the mediastinum was associated with substantial thin, vascular hyperplasty in the circumference, so abrasions could have caused bleeding. We were able to avoid such bleeding by performing exfoliation while cauterizing the lesion with a supersonic wave coagulotomy device. After stopping any bleeding, we detained a 19-French BLAKE™ silicone drain tube and sutured the wound.

After moving the patient into the dorsal position, the otolaryngologists performed their operation. They first made a 10-cm collar incision at the patient’s neck. They cut the platysma muscle, exfoliated the tumor from the anterior cervical muscles, and transected it from the left lobe before resecting the swollen right lobe of the thyroid gland and confirming the recurrent laryngeal nerve with a nerve stimulator (Fig. 2D). After confirming that there were no malignant findings in the tumor by an intraoperative frozen section diagnosis, they placed a drain and closed the wound to complete the operation.

After the surgery, the patient was extubated, and her vocal cords were confirmed not to be constricted under a laryngeal mask. The patient was then awakened from general anesthesia. The total operation time was 5 h and 20 min (thoracoscopic surgery time: 1 h 23 min, changing position and intubation tube exchange time: 41 min, cervical surgery time: 3 h 16 min), and the total amount of bleeding was 130 g. In this way, resection of the goiter of the right lobe was performed with thoracoscopic assistance.

**Fig. 3:**
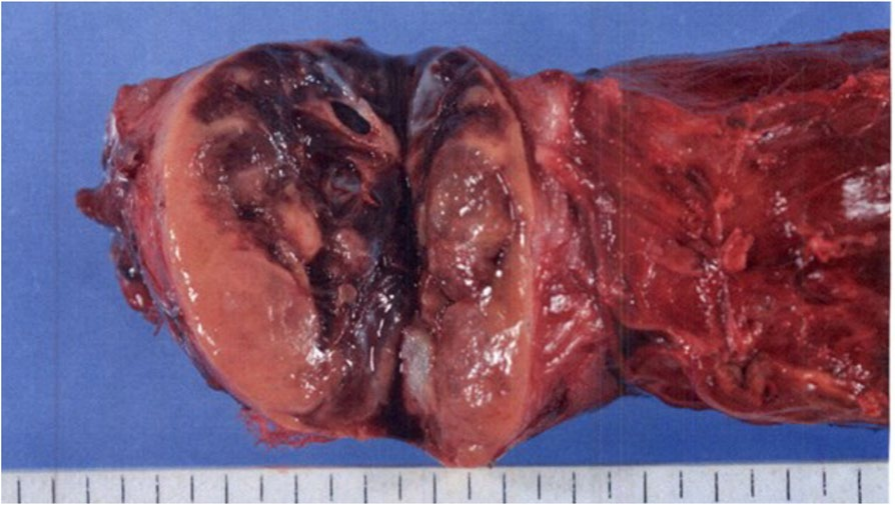
Findings of the resected specimen. The resected specimen measured 150 × 52 × 48 mm. The cut surface was solid yellow, and the tumor included some cysts

**Fig. 4:**
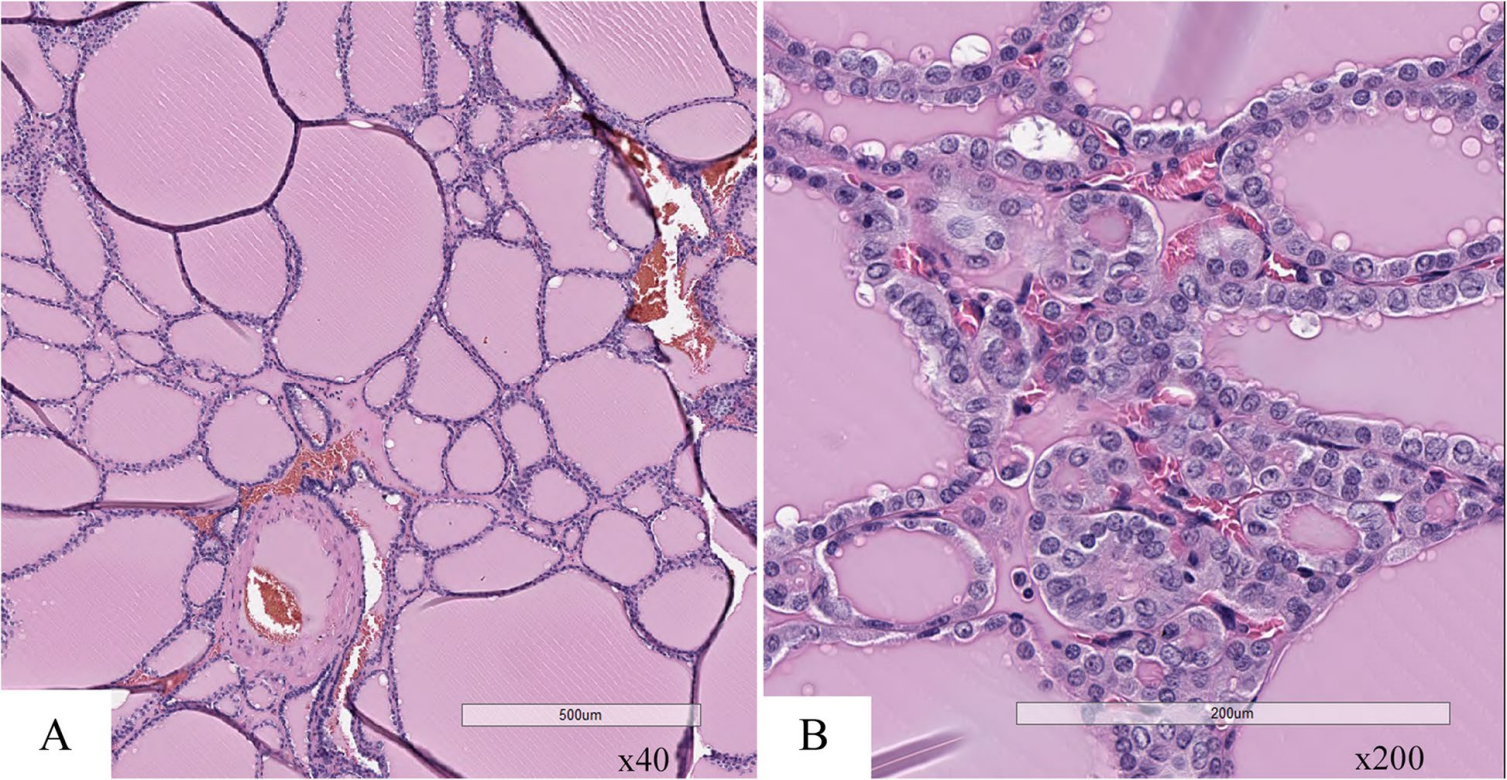
Histopathology findings. **A** A histopathological examination revealed various-sized follicles in low-power fields. **B** No malignant cells were found in epithelium cells in high-power fields

The resected specimen measured 150 × 52 × 48 mm. The cut surface was solid yellow, and the tumor included some cysts (Fig. 3). A histopathological examination revealed various-sized follicles in low-power fields (Fig. 4A), and no malignant cells were found in the epithelium cells in high-power fields (Fig. 4B). The pathologist diagnosed the tumor as a benign thyroid goiter.

**Fig. 5:**
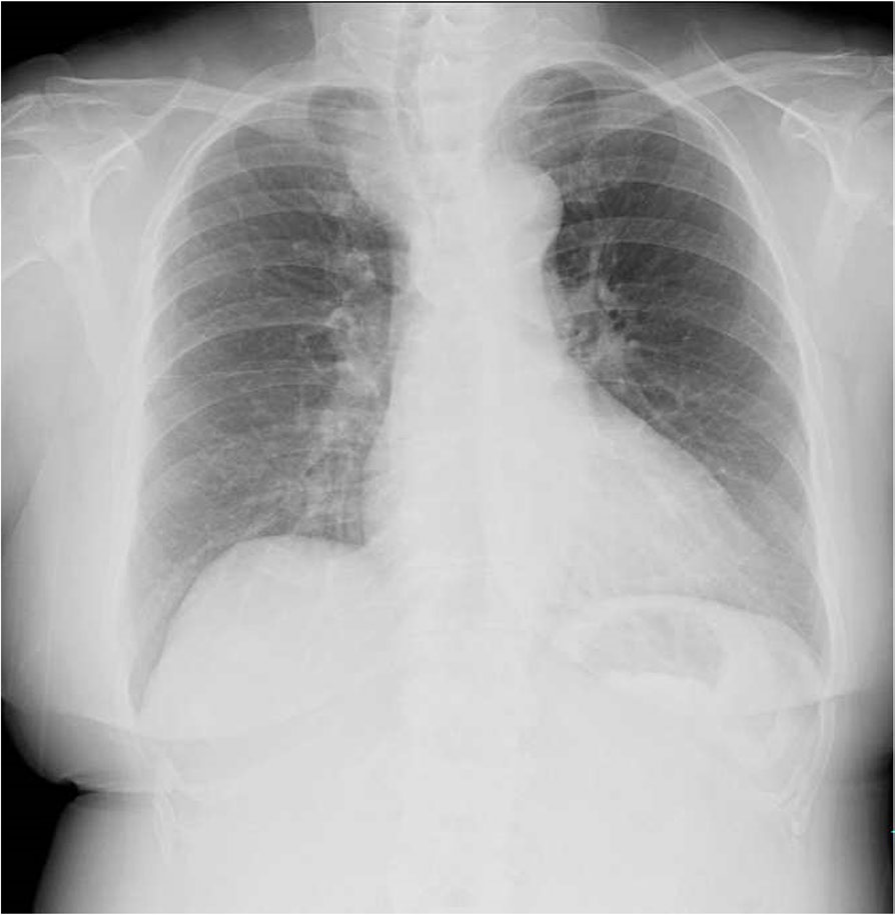
Postoperative chest X-ray findings. The tracheal stenosis had improved at 1 month after the surgery

The patient’s postoperative course was uneventful except for slight right recurrent nerve paralysis, and she left the hospital on day 8 after the operation. Chest X-ray revealed the improvement of tracheal stenosis (Fig. 5). The trachea was shifted to the right side due to the remaining goiter on the left side. Regarding the left-side goiter, the thyroid function was normal, and there were no findings suggesting malignancy or airway pressure symptoms, so we decided to perform follow-up at the otolaryngology department. She had no evidence of recurrence of dyspnea symptoms at the time of this writing.

## Discussion

Wakely C. P. G. et al. classified intrathoracic goiter into three types: small substernal extension (80%), where the main part of the mass is in the neck; partial intrathoracic (15%), where the main part of the mass is in the thoracic cavity; and complete intrathoracic (5%), where all of the mass is in the thoracic cavity [2]. Crescenzo V. D. et al. reported on 97 patients with cervicomediastinal goiters. Of these patients, 55 (57%) had respiratory manifestations, 22 (23%) had dysphasia, 18 (18.5%) had hyperthyroidism, 18 (18.5%) had tachycardia, 9 (9.2%) had anxiety, 6 (6.1%) had weight loss, 4 (4.6%) had hyperhidrosis, 4 (4.6%) had tremors, 9 (9.2%) had dysphonia, and 6 (6.1%) had superior vena cava syndrome. However, 13 (13.8%) had no symptoms at all [3].

Regarding the surgical indications for substernal goiter, Liang S. W. reported that surgical resection should be considered even for elderly patients because of the risks of mass compression symptoms (dyspnea and dysphasia) and malignancy and the low morbidity rate of surgery [4]. The present patient had symptoms of dyspnea due to compression of the trachea by the goiter. Therefore, this was a suitable case for surgery adaptation.

Our patient’s goiter was not finally diagnosed as a malignant tumor pathologically. However, regarding risks of malignancy, Yamashita H. et al. reported that 256 (30.7%) of 835 patients with adenomatous goiter > 10 mm in size had thyroid cancer [5].

Most cervicomediastinal goiters are situated in the anterior mediastinal compartment. In the present case, the cervicomediastinal goiter extended to the bronchial bifurcation in the middle mediastinum. Goiter extending beyond the aortic arch is difficult to extract by a cervical approach alone, often requiring an intrathoracic approach, such as manubriotomy or sternotomy [6]. Kasuga Y. et al. reported that goiters extending > 6 cm below the articulatio sternoclavicularis in the anterior mediastinum or > 5 cm below in the posterior mediastinum were suited for sternotomy [7]. In the present case, the tip of the goiter was 7 cm below the articulatio sternoclavicularis, extending to the carina level, making it suitable for sternotomy.

Several different types of sternotomy have been developed. Median sternotomy is recommended in cases of malignant tumors invading the brachiocephalic vein. The L-shaped route is used for longitudinal sternotomy and lateral sternotomy at the 4th intercostal space and is recommended for tumors with extensive hilar invasion. The T-shaped route is used for longitudinal sternotomy and neck collar incision and is recommended for tumors located around the trachea in the upper mediastinum [8]. Hasumi T. et al. reported that they were able to resect a benign tumor that occurred in the cervicothoracic border region (C7–Th4) with a lower end extending to the aortic arch or the superior border of the azygos vein with a good field of view by performing a supraclavicular neck collar incision and partial sternotomy (L or inverted L-shaped mini-sternotomy) [9]. In the present case, no hilar infiltration was observed. However, the lower edge of the goiter exceeded the level of the 4th thoracic vertebra and was outside the upper mediastinum. No infiltration was observed, and median sternotomy can be highly invasive and lead to complications, such as bleeding, infection, dysraphism of the sternum, and pain. Rebei M. et al. compared the outcomes of anterior mediastinal tumor resection performed via VATS in 91 cases and sternotomy in 102 cases. Of the 102 patients who underwent sternotomy, 6 had complications of infection, 5 had myasthenia gravis, 2 had bleeding complications, and 3 died. In contrast, among the 91 patients who underwent VATS, the only complication was atelectasis in 2 patients. The average length of hospital stay was 6 days for sternotomy and 4 days for VATS. The authors thus concluded that VATS had improved efficacy with better outcomes and a shorter hospital stay than sternotomy with similar results [10].

However, when the goiter is diagnosed as or presumed to be malignant, abrasion under thoracoscopy should be avoided. Magistrelli P. et al. noted that, in select cases (generally malignancies with local infiltration of mediastinal soft tissues and adhesions to large vessels), split sternotomy may be a safer approach than video-assisted thoracic surgery (VATS) without increasing the morbidity [11]. Shigemura N. et al. reported the application of VATS with a supraclavicular window in five high-risk patients with huge anterior mediastinal goiters, resulting in an uncomplicated postoperative course and favorable outcomes in all cases [12]. Bhargav P. R. et al. recently reported a series of posterior mediastinal goiters (11 cases) that were treated through a thoracoscopic approach [13]. Our method is similar to that described in their report, although they placed working ports at higher intercostal spaces than we did (2nd and 4th) and used CO_2_ insufflation at 6 mmHg during the VATS operation. In the present case, we approached from a lower intercostal space and were able to secure a good working space, and the backside of the brachiocephalic and azygos veins was visible and able to be safely dissected.

Landerholm K. et al. reported the independent risk factors for recurrent laryngeal nerve paralysis after benign thyroid surgery to be intrathoracic goiter (odds ratio [OR], 3.57; 95% confidence interval [CI], 1.70–7.48), ipsilateral redo surgery (*OR*, 3.64; 95% *CI*, 1.00–13.28), and total lobectomy (*OR*, 2.41; 95% *CI*, 1.05–5.55) [14]. In our case, the right recurrent laryngeal nerve was identified during the operation and preserved, but right vocal cord paralysis still occurred postoperatively. The nerve had been hyperextended by the goiter extending into the thoracic space, and the dissecting distance was long. That was thought to increase the risk of injury during surgery and postoperative paralysis. In order to prevent damage to the recurrent laryngeal nerve, early surgery before hyperstretching of the recurrent laryngeal nerve occurs, and gentle operation was considered. Lin Y. S. et al. reported a retrospective cohort study of 2104 patients who underwent resection of substernal goiters. Transient recurrent laryngeal nerve injury was observed in 17 (12.1%) patients, whereas permanent recurrent laryngeal nerve injury with unilateral vocal cord palsy developed in 6 (4.3%) patients. The incidence of recurrent laryngeal nerve injury was 4.7% and was more common with right-sided goiters than left-sided ones, possibly because of anatomic variations. During substernal goiter development into the mediastinum, the normal route of the recurrent laryngeal nerve may be altered by a space-occupying lesion, with a greater impact on the right side than on the left side. Therefore, the right recurrent laryngeal nerve may be more susceptible to damage during substernal goiter surgery than the left one [15].

## Conclusion

A giant cervicomediastinal goiter extending to the bifurcation of the trachea was safely resected through a cervical collar incision and VATS approach, which enabled the patient to be discharged from the hospital with a good quality of life.

## Data Availability

All data generated or analyzed in this study are included in this manuscript.

## Abbreviations

CT: Computed tomography
CO2: Carbon dioxide
OR: Odds ratio
VATS: Video-assisted thoracic surgery
